# Neuroimaging Mechanism of Cognitive Behavioral Therapy in Pain Management

**DOI:** 10.1155/2022/6266619

**Published:** 2022-02-02

**Authors:** Shangyi Bao, Mengyuan Qiao, Yutong Lu, Yunlan Jiang

**Affiliations:** ^1^Department of Hematology, Hospital of Chengdu University of Traditional Chinese Medicine, Chengdu, Sichuan, China; ^2^Department of Geriatrics, Hospital of Chengdu University of Traditional Chinese Medicine, Chengdu, Sichuan, China; ^3^School of Nursing, Chengdu University of Traditional Chinese Medicine, Chengdu, Sichuan, China; ^4^Department of Nursing, Hospital of Chengdu University of Traditional Chinese Medicine, Chengdu, Sichuan, China

## Abstract

*Purpose.* To review the recent neuroimaging studies on cognitive-behavioral therapy (CBT) for pain management, with the aim of exploring possible mechanisms of CBT. *Recent Findings*. Current studies can be divided into four categories, mixed pain, fibromyalgia, migraine, and experimental pain, based on the type of disease included, with the same or different changes of brain regions after CBT intervention. According to structural and functional MRI analyses, changes of brain gray matter volume, activation and deactivation of brain regions, and intrinsic connectivity between brain regions were observed after CBT sessions. The brain regions involved mainly included some areas related to cognitive and emotional regulation. After comparison, the DLPFC, OFC, VLPFC, PCC and amygdala were found to be recurrent in multiple studies and may be key regions for CBT intervention in pain management. In the treatment of mixed chronic pain, CBT may decrease the gray matter volume of DLPFC, reduce ICN connection of OFC within the DAN network, and increase fALFF of the PCC. For FM intervention, CBT may activate the bilateral OFC and VLPFC, while in migraine, only the right OFC, VLPFC, and DLPFC were found to be more activated after CBT. In addition, the differential action of the left and right amygdala has also been shown in the latest study of migraine. In heat-evoked pain, CBT may increase the deactivation of the PCC, the connectivity between the DMN and right VLPFC, while diminishing the deactivation of VLPFC. *Summary*. After CBT, the brain showed stronger top-down pain control, cognitive reassessment, and altered perception of stimulus signals (chronic pain and repeated acute pain). The DLPFC, OFC, VLPFC, PCC, and amygdala may be the key brain regions in CBT intervention of pain.

## 1. Introduction

Cognitive-behavioral therapy (CBT) came out in the 1960s, which is a structured psychotherapeutic intervention that targets maladaptive cognitive factors to reduce negative affect [1]. Since then, it has been extensively used in the treatment of psychiatric disorders, such as depression, anxiety disorders, and personality disorders [2]. In recent years, numerous studies have demonstrated its application value in nonpsychiatric disorders, including irritable bowel syndrome, insomnia, and chronic pain conditions, such as migraine and fibromyalgia. CBT is available for all ages, from children to the elderly, and the treatment modality has evolved from one-on-one communication to team therapy, from face-to-face communication to telephone therapy, and the newly explored online therapy. It can be seen that CBT is a treatment with great clinical application value.

In recent years, pain, the fifth vital sign, has developed into a global problem [3]. Chronic pain can even last for decades, severely affecting physical and mental health. The importance of nonpharmacological treatment of chronic pain has become increasingly significant due to problems such as addiction to painkillers. Pain and its neural representation are highly affected by cognitive factors [4–6]. Clinical studies on CBT and chronic pain have proliferated. With the development of neuroimaging techniques, it has been increasingly used to conduct studies about CBT to explore the mechanisms of CBT for pain management. In this review, we intend to review the recent neuroimaging studies on CBT for pain management, with the aim of exploring possible mechanisms of CBT, improving the CBT process to increase clinical efficacy, and providing a basis for reversing chronic pain in the future.

## 2. Method

In September 2021, we searched PubMed and Web of Science. We searched the mentioned database using search terms including “CBT” AND “pain,” “CBT” AND “fMRI” AND “pain,” “CBT” AND “pain” AND “imaging,” “cognitive” AND “pain” AND “imaging,” and “cognition” AND “pain” AND “imaging.” We mainly selected literature from 2016 to 2021. In addition, the early literature that were frequently cited and of high value were also cited. The reference lists for included studies were manually screened by members to minimize the omission of potentially eligible articles.

## 3. Structural and Functional Changes of the Brain due to CBT

In recent 2 years, a large number of clinical studies had taken CBT as a management measure of different kinds of pain, including mixed unlocated chronic pain [7–18], back pain [19, 20], low back pain [21–26], chronic pancreatitis [27], fibromyalgia [28, 29], functional abdominal pain [30], trigeminal neuralgia [31], haemophilia pain [32], osteoarthritis pain [33–35], perioperative pain [36–39], orofacial pain [40], diabetic peripheral neuropathic pain [41], and provoked vestibulodynia [42]. Previous studies on structural changes in the brain of patients with chronic pain indicated the presence of neuroplasticity in areas associated with the experience and anticipation of pain [43]. In the past few years, there has been growing interest in studying changes in brain structure and connectivity after CBT interventions for pain to explore the underlying mechanism. Previous studies of the neuroimaging mechanism can be classified according to the type of pain enrolled, including mixed diagnosis and pain with clear diagnosis and experimental irritation.

### 3.1. Mixed Diagnosis of Pain

Several studies have shown a correlation between gray matter (GM) reduction in some regions (including volume or density) and the duration or intensity of pain [44–48]. According to former research studies, Seminowicz et al. [49] conducted a study that enrolled 26 patients (chronic pain (CP), *n* = 13; healthy controls (HC), *n* = 13). Patients in the CP group received 11 times, 90-minute weekly CBT group sessions, and were scanned twice by anatomical MRI before and after these CBT sessions. Voxel-based morphometry (VBM) analysis was conducted on MRI data. After CBT sessions, results showed that GM volume (GMV) in the bilateral dorsolateral prefrontal cortex (DLPFC), posterior parietal cortex (PPC), and some other sensory, motor, and affective areas increased, while GMV in the left supplementary motor area (SMA) reduced. They also found that increased GMV in prefrontal and parietal areas was related to decreased pain catastrophizing, which is regarded as an important target for the treatment of CP in the latest research [15]. These results suggested that after CBT, the brain has a stronger top-down control of pain and a cognitive reassessment of pain and a change in the perception of noxious signals. Notably, in this early study, they rigorously performed analyses on the exclusion of depression and natural changes of GM density (GMD) across time. These factors are not taken into account in many later studies.

Shpaner et al. [50] conducted a randomized control trial (RCT) and enrolled 38 participants with chronic musculoskeletal pain, who were divided into the CBT group (*n* = 19) and educational materials group (EDU, *n* = 19). They observed changes in intrinsic functional connectivity (iFC) of the brain after 11 weeks of CBT by using functional MRI (fMRI). The results showed that after CBT, iFC between the anterior default mode network (DMN) and amygdala/periaqueductal gray (PAG) decreased, which was related to the prepost change in self-efficacy for coping with symptoms (*ρ* = −0.329, *P*=0.044). And, iFC between the basal ganglia (BG) network and right secondary somatosensory (S2) cortex increased, which was revealed to the decrease in pain symptoms (*ρ* = −0.343, *P*=0.035) and the increase in other clinical results such as self-efficacy for pain management (*ρ* = 0.574, *P* < 0.001) [50]. CBT patients also had increased posttherapy fractional amplitude of low-frequency fluctuations (fALFF) in the bilateral posterior cingulate cortex (PCC) and the cerebellum. In addition, they examined the possible confounding influence of medication and menstrual cycle.

Yoshino et al. [51] used resting-state functional magnetic resonance imaging (R-fMRI) to examine neural changes after CBT (CP, *n* = 29; age-matched HC, *n* = 30). After a weekly 12-session CBT, abnormal intrinsic connectivity network (ICN) connections in CP patients normalized, including the orbitofrontal cortex (OFC), inferior parietal lobule within the dorsal attention network (DAN), and the paracentral lobule within the sensorimotor network. Interestingly, inspired by the previous studies on posttreatment prediction [52, 53], they also conducted relevant experiments. Among them, higher ICN connection strength in OFC was associated with a greater decrease in pain intensity. The lower ICN connectivity strength in the dorsal posterior cingulate cortex within the DAN was related to positive CBT-related clinical improvements.

### 3.2. Fibromyalgia

The study of Jensen et al. [54] is the earliest published neuroimaging study on the therapeutic mechanisms of CBT for chronic pain, which was reported in 2012. This randomized, 12-week, waiting-list controlled clinical trial enrolled 43 female participants with fibromyalgia (FM) syndrome (CBT *n* = 25; controls *n* = 18). FMRI during pressure-evoked pain was assessed twice before and after 12-week CBT. The analysis showed that CBT activations in the ventrolateral prefrontal cortex (VLPFC) and lateral orbitofrontal cortex increased, which were associated with executive cognitive control. The change in anxiety was significantly positively related to the VLPFC activation (*r* = 0.67, *P* < 0.05, 2-tailed). In addition, they found that coherence between the VLPFC and thalamus was increased. In former studies, thalamic activity was decreased in FM [52, 55, 56] and other CP conditions [57]. So, it was suggested that CBT may also influence the thalamus and other lower structures of the brain.

Shipman [58] enrolled 16 high-catastrophizing FM patients (CBT, *n* = 8; EDU, *n* = 8). An innovation over previous studies is that they performed a total of three times fMRI scans, at baseline, posttreatment, and 6-month follow-up, in order to observe the persistence outcomes of CBT. The result showed that resting state connectivity between the primary somatosensory cortex (S1) and anterior/medial insula was reduced after CBT, which was correlated with concurrent treatment-related reductions in catastrophizing [59]. Furthermore, a clear potential sequential association was shown in this study. Changes in catastrophizing and insula-S1 connectivity occurred after the 1-month CBT sessions, while the pain interference changed significantly at 6-month follow-up. This sequential association provided us with the potential for fMRI to be used as an early marker tool to identify the benefits of long-term treatment.

Interestingly, McCrae et al. [60] had compared the changes of nerve activation in pain response after traditional CBT for pain (CBT-P) and CBT for insomnia (CBT-I). They enrolled 32 patients with FM who underwent an experimental pain protocol during fMRI before and after CBT-P or CBT-I or waitlist control period. The fMRI analysis indicated that 12 regions showed significant interactions after CBT intervention. Activation decreased in 8 regions after CBT-I and in 3 regions after CBT-P, which was assessed by blood oxygen level-dependent (BOLD) response to pain. The better sleep improvement from CBT-I may account for this difference. Later, they conducted the latest study which innovatively regarded arousal and insomnia as mediating mechanisms in CBT for pain. McCrae et al. [61] enrolled 130 female participants with comorbid FM and insomnia, and carried out direct interventions with CBT-I. Similar to the design of Lazaridou et al., they planned structural and functional MRI scans 4 times to observe the persistence effects. They focused on central sensitisation (CS), which is an important character of FM [62, 63]. They proposed that CBT-I had the effect of reducing arousal, improving sleep, and reversing the negative hypothalamic-pituitary-adrenal (HPA) and central nervous system (CNS) changes (i.e., reversing CS) that sustain CP [61]. The data of this RCT have not been collected yet; it is believed that the publication of the full trial results will provide a deeper understanding of the intervention mechanism of CBT-I.

### 3.3. Migraine

Unlike most current CP research studies, Nahman-Averbuch et al. [64] recruited 18 adolescents with migraine in the clinical trial (15 females, age 15.1 ± 2.1 years). These adolescents underwent an 8-week CBT with their parents. The results showed a decrease in headache frequency from 15 ± 7.4 per month to 10 ± 7.4 per month after CBT sessions (*P* < 0.001). Similar to former studies, they found changes both in brain activation and functional connectivity. According to fMRI analysis, CBT resulted in activation of the OFC, VLPFC, and DLPFC regions on the right side of the brain, increased connectivity between the amygdala and paracingulate gyrus, PFC, and occipital cortex, but led to bilateral deactivation of the cerebellum. Additionally, the reduction in headache was correlated with bilateral activation of the occipital cortex, lingual gyrus, angular gyrus, and superior parietal lobule.

Interestingly, in this study, headache reduction was associated with opposite changes in left and right amygdala connectivity. The decrease in headache after CBT was associated with increased connectivity between the left amygdala and the occipital cortex and the reduced connectivity between the right amygdala and the paracingulate gyrus and DLPFC [64]. The amygdala is regarded as a key region in nociceptive processing and is highly connected to other pain regions such as the PFC, thalamus, anterior cingulate cortex, insula, and PAG [65, 66]. Changes in the amygdala function and structure have also been found in previous studies in adults suffering from migraine [67–69]. The amygdala and PFC are structurally and functionally related [70–73], and the amygdala has an inhibitory action and can disable the activity of MPFC [66, 74, 75]. This finding indicated that the left and right amygdala may have different roles in pain processing. It is suggested that CBT decreased the compensatory action of the right amygdala on the DLPFC. While the antinociceptive action of the left amygdala on the dorsal medial prefrontal cortex (DMPFC) was increased after CBT.

### 3.4. Experimental Pain

In contrast to the above studies that recruited patients, Kucyi et al. [76] recruited 30 healthy participants (CBT *n* = 17; control *n* = 13) to undergo equal amounts of heat-evoked pain, and they used the identical subjective reported pain levels before and after treatment. The fMRI analysis showed that there were pain-evoked deactivations in regions of the default mode network (DMN), including the bilateral posterior cingulate cortex (PCC)/precuneus (PCU), medial prefrontal cortex (MPFC), and lateral parietal cortex (LPC). There were no statistically significant group differences before intervention (*P* < 0.05), but after the intervention, the deactivation was significantly lower in the control group compared to the CBT group (*P* < 0.05). This means that repeated pain exposure eradicated DMN deactivation in the nonintervened ones but CBT could reverse the effect. Moreover, reduced deactivation of the right ventrolateral prefrontal cortex (VLPFC) of the executive control network and increased spontaneous functional connectivity between the DMN and right VLPFC was observed in the CBT group. Former studies suggested that changes in MPFC activity and connectivity were related to development of CP [77, 78]. In addition, patients with CP showed a lack of DMN deactivation during painful stimulation, which was recovered after successful analgesic treatment [79]. It can be seen that chronic or repeated acute pain exposure could lead to decreased pain-induced DMN deactivation, but this decline can be prevented/reversed by CBT or analgesic treatment.

## 4. Discussion

**Figure 1:**
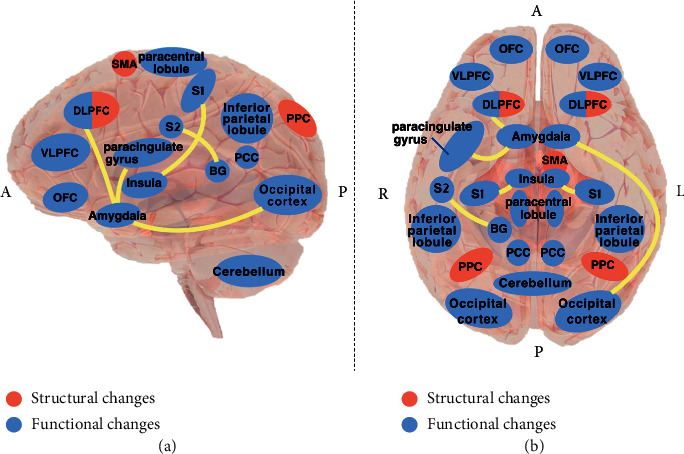
Illustration of the main mechanisms of CBT in pain management. (a) Side perspective view. (b) Bottom perspective view. Abbreviations are annotated after Table 1.

The aforementioned studies showed that CBT may relieve mixed chronic pain, fibromyalgia, migraine, and heat-stimulated pain by causing structural or functional changes in multiple brain regions. The main mechanisms of CBT are given in Table 1 and Figure 1. Previous studies mainly used Brodmann area for brain region partitioning; still, some also used anatomical automatic labelling (AAL), which may lead to crossover in the results. As given in Table 1, some key regions were repeatedly observed in several studies, including the DLPFC, OFC, VLPFC, PCC, and amygdala.

DLPFC was the only region showing both functional and structural alterations, suggesting that CBT may relieve mixed pain by decreasing GMV of DLPFC, and be therapeutic for migraine by activating the right DLPFC and weakening its association with the right amygdala. OFC and VLPFC had also shown their importance in the mechanism of CBT-P, which are adjacent to DLPFC. The findings suggested that CBT may treat FM and migraine by activating the right or bilateral OFC and VLPFC. In addition, CBT may reduce ICN connection of OFC within the DAN network in mixed pain treatment and reduce deactivation of VLPFC and enhance its connectivity with the DMN in heat-evoked pain intervention. Moreover, CBT may relieve mixed pain by reducing iFC between the medial prefrontal cortex and the amygdala, which is an important component of the anterior DMN. The reduction in the volume of SMA and the decreased activation of the MFG and IFG may also be associated with the relief of mixed pain or FM due to CBT.

Functional changes in the PCC, cingulate gyrus, and paracingulate gyrus were also found in the above four types of pain studies. It is suggested that CBT may treat mixed pain by increasing fALFF of the PCC, relieve heat-evoked pain by increasing its deactivation, intervene in FM by reducing the activation of the right cingulate gyrus, and treat migraine by reducing the association of the paracingulate gyrus with the right amygdala.

The differential action of the left and right amygdala has gradually emerged as research progresses. While earlier studies showed a decrease in iFC of the amygdala and the anterior DMN due to CBT, the latest study showed that the left and right amygdala had opposed alterations in connectivity with other regions. It is implied that CBT-P may treat migraine by increasing the connectivity between the left amygdala and the occipital lobe and decreasing the connectivity between the right amygdala and the paracingulate gyrus and DLPFC. The amygdala and the lentiform nucleus are important components of the BG. The therapeutic effects of CBT may also be associated with enhanced iFC between the BG network and the right S2 as well as diminished activation of the right lentiform nucleus.

The following problems exist in the published studies. (1) Several studies enrolled patients with mixed diagnoses of pain. Small number of subjects led to the lack of subgroup analysis of each diagnosis. Different types of pain may cause different central imaging changes, and the therapeutic effects of CBT may be different. (2) Only a few studies had considered the mediating role of insomnia, medication, menstrual cycle, depression, and other factors. These neuroimaging changes after CBT may be confounded with the performance of mood and insomnia improvement, and the causal relationship is difficult to clarify. (4) Only a few studies controlled or matched for pain levels. It is speculated that the therapeutic effect of CBT may be associated with pain severity, based on the current clinical study findings. Published studies had not shown changes in the brain areas in patients with ineffective clinical symptom improvement, which are common in the real world, and lacked subgroup analyses of different pain levels.

Future trend: (1) in the future, subgroup analysis of different types of pain should be designed to reduce the confounding factors of different types of pain. (2) Future research needs to reduce the mixed effects of insomnia and emotions (depression and anxiety). (3) fMRI can be used to predict the curative effect of CBT according to the level and stage of pain and the structural connection state of the inherent brain region. (4) Neuroimaging studies can be designed to make improvements of CBT. It is possible to refine whether patients with certain clinical characteristics can benefit specifically after strengthening some stages of the CBT session. Neuroimaging studies can guide innovation in the form of CBT by comparing the effects of online-offline and individual-team brain stimulation.

## 5. Conclusions

Current studies of neuroimaging mechanisms of CBT used structural and functional MRI to analyze changes in brain gray matter volume, activation and deactivation of brain regions, and intrinsic connectivity between brain regions. The involved networks contained the DMN, DAN, and sensorimotor network. Pain is a multidimensional sensory and emotional experience associated with anxiety, depression, and insomnia. The current findings indicated that many brain regions responsible for cognition and emotion were involved in the mechanism of CBT, including the frontal cortex, parietal cortex, occipital cortex, somatosensory cortex, basal ganglia, amygdala, cerebellum, insula and cingulate gyrus. After CBT, the brain showed stronger top-down pain control, cognitive reassessment, and altered perception of stimulus signals (chronic pain and repeated acute pain). In order to get more accurate results in future studies, separate analyses for a specific type of pain could be considered to rule out the influence of mixed factors such as different kinds of pain, anxiety, depression, and insomnia.

## Figures and Tables

**Table 1:** Comparison of findings on neuroimaging changes in CBT for pain-related disorders.

Type	Journal year	L/R	Regions	Structural changes	Functional changes	+/−	References
Mixed	2013	B	**DLPFC** ^*∗*^, PPC	GMV	—	−	Seminowicz et al. [49]
		L	SMA	GMV	—	−	
	2014	B	Anterior **DMN**^*∗*^ and the **amygdala**^*∗*^/PAG	—	iFC	−	Shpaner et al. [50]
		R	BG network and the right S2	—	iFC	+	
		B	**PCC** ^*∗*^, the cerebellum	—	fALFF	+	
	2018	B	**OFC** ^*∗*^ within the **DAN**^*∗*^	—	ICN connection	−	Yoshino et al. [51]
			IPL within the **DAN**^*∗*^ and PCL within the sensorimotor network	—	ICN connection	+	

FM	2012	B	**VLPFC** ^*∗*^, **OFC**^*∗*^	—	Activation	+	Jensen et al. [54]
	2016	B	S1 and anterior/medial **insula**^*∗*^	—	Connectivity	−	Lazaridou et al. [59]
	2021	B	STG, IFG	—	Activation	−	McCrae et al. [60]
		R	**Insula** ^*∗*^, MOG, lentiform nucleus, **cingulate gyrus**^*∗*^	—	Activation	−	
		L	ANG, MFG, IOG, MTG	—	Activation	−	

Migraine	2020	R	**OFC** ^*∗*^, **VLPFC**^*∗*^, **DLPFC**^*∗*^	—	Activation	+	Nahman-Averbuch et al. [64]
		L	The left **amygdala**^*∗*^ and the occipital cortex	—	Connectivity	+	
		R	The right **amygdala**^*∗*^ and the paracingulate gyrus and **DLPFC**^*∗*^	—	Connectivity	−	

Heat-evoked	2016	B	**PCC** ^*∗*^, PCU, MPFC, LPC	—	Deactivation	+	Kucyi et al. [76]
		B	**VLPFC** ^*∗*^	—	Deactivation	−	
		R	**DMN** ^*∗*^ and right **VLPFC**^*∗*^	—	Connectivity	+	

The bolded and asterisked markers mean that the brain region was repeatedly mentioned in multiple studies. L: left; R: right; B: bilateral; GMV: gray matter volume; iFC: intrinsic functional connectivity; fALFF: fractional amplitude of low-frequency fluctuations; ICN: intrinsic connectivity network; DLPFC: dorsolateral prefrontal cortex; PPC: posterior parietal cortex; SMA: supplementary motor area; DMN: default mode network; PAG: periaqueductal gray; BG: basal ganglia; S2: the secondary somatosensory cortex; PCC: posterior cingulate cortex; OFC: orbitofrontal cortex; DAN: dorsal attention network; IPL: inferior parietal lobule; PCL: paracentral lobule; VLPFC: ventrolateral prefrontal cortex; S1: the primary somatosensory cortex; STG: superior temporal gyrus; IFG: inferior frontal gyrus; MOG: middle occipital gyrus; ANG: angular gyrus; MFG: middle frontal gyrus; IOG: inferior occipital gyrus; MTG: middle temporal gyrus; PCU: precuneus; MPFC: medial prefrontal cortex; LPC: lateral parietal cortex.
